# Investigating Origins of FLIm Contrast in Atherosclerotic Lesions Using Combined FLIm-Raman Spectroscopy

**DOI:** 10.3389/fcvm.2020.00122

**Published:** 2020-07-21

**Authors:** Julien Bec, Tanveer Ahmed Shaik, Christoph Krafft, Thomas W. Bocklitz, Alba Alfonso-Garcia, Kenneth B. Margulies, Jürgen Popp, Laura Marcu

**Affiliations:** ^1^Department of Biomedical Engineering, University of California, Davis, Davis, CA, United States; ^2^Institute of Physical Chemistry and Abbe Center of Photonics (IPC), Friedrich-Schiller-University, Jena, Germany; ^3^Leibniz Institute of Photonic Technology, Jena, Germany; ^4^Cardiovascular Institute, Perelman School of Medicine, University of Pennsylvania, Philadelphia, PA, United States

**Keywords:** imaging, spectroscopy, atherosclerosis, Raman spectroscopy, FLIm, time resolved fluorescence

## Abstract

**Background:** Fluorescence lifetime imaging (FLIm) is a spectroscopic imaging technique able to characterize the composition of luminal surface of arterial vessels. Studies of human coronary samples demonstrated that distinct atherosclerotic lesion types are characterized by FLIm features associate with distinct tissue molecular makeup. While conventional histology has provided indications about potential sources of molecular contrast, specific information about the origin of FLIm signals is lacking. Here we investigate whether Raman spectroscopy, a technique able to evaluate chemical content of biological samples, can provide additional insight into the origin of FLIm contrast.

**Methods:** Six human coronary artery samples were imaged using FLIm (355 nm excitation)-Raman spectroscopy (785 nm excitation) via a multimodal fiber optic probe. The spatial distribution of molecular contrast in FLIm images was analyzed in relationship with histological findings. Raman data was investigated using an endmember technique and compared with histological findings. A descriptive modeling approach based on multivariate regression was used to identify Raman bands related with changes in lifetime in four spectral channels (violet: 387/35 nm, blue: 443/29 nm, green: 546/38 nm, and red: 628/53 nm).

**Results:** Fluorescence lifetime variations in the violet, blue and green spectral bands were observed for distinct areas of each tissue sample associated with distinct pathologies. Analysis of Raman signals from areas associated with normal, pathological intimal thickening, and fibrocalcific regions demonstrated the presence of hydroxyapatite, collagenous proteins, carotene, cholesterol, and triglycerides. The FLIm and Raman descriptive modeling analysis indicated that lifetime increase in the violet spectral band was associated with increased presence of cholesterol and carotenes, a new finding consistent with LDL accumulation in atherosclerotic lesions, and not with collagen proteins, as expected from earlier studies.

**Conclusions:** The systematic, quantitative analysis of the multimodal FLIm-Raman dataset using a descriptive modeling approach led to the identification of LDL accumulation as the primary source of lifetime contrast in atherosclerotic lesions in the violet spectral range. Earlier FLIm validation studies relying on histopathological findings had associated this contrast to increased collagen content, also present in advanced lesions, thus demonstrating the benefits of alternative validation methods.

## Introduction

Atherosclerotic cardiovascular diseases is a major cause of mortality (1). Despite decades of studies, the mechanisms behind atherosclerotic plaque progression leading to acute coronary events are not fully understood. Conventional imaging techniques, such as intravascular ultrasound (IVUS) and intravascular optical coherence tomography (iv-OCT) have enabled *in vivo* assessment of atherosclerotic lesions morphology, but they lack sensitivity to associated compositional features. Thus, more recent efforts are focused on developing complementary label-free imaging techniques able to detect compositional changes associated with morphological changes and lesion progression (2). Among these are intravascular declinations of near-infrared spectroscopy (NIRS) (3), photoacoustic (PA) (4), polarization sensitive OCT (PS-OCT) (5), fluorescence spectroscopy (6), and Raman spectroscopy (RS) (7). NIRS, PS-OCT, and PA enable the identification of features from advanced lesions such as large lipid pools and cholesterol crystals, but the assessment of compositional changes associated with early lesion development, a key factor to further the understanding of lesion pathogenesis, is still elusive.

The autofluorescence signature of arterial vessels has shown potential for the identification of compositional changes associated with lesion development (8). Imaging catheters combining fluorescence lifetime imaging techniques with IVUS and OCT have been recently reported and found suitable for the interrogation of coronary arteries (6, 9). However, autofluorescence signal from biological tissues is complex as several fluorophores contribute to the emission (10). Fluorophores present peak emissions at specific wavelengths, and therefore may in some cases be identified using spectral characteristics. Unfortunately, the wide emission bands typical of biological fluorophores hinder identification of individual fluorophores based on spectral characteristics. This is especially true for the violet region where several common molecules present fluorescence emission peaks (collagen, elastin, lipids, lipoproteins, and proteoglycans). Time-resolved measurements enable further discrimination, but identification of specific fluorophores still presents challenges: comparison of the spectroscopic signature of tissue with measurements of pure compounds has been reported for intensity based approaches (11), but this approach is not suitable for fluorescence lifetime, as lifetime characteristics of molecular species are heavily influenced by their microenvironment (12), and therefore measurements of pure compounds in solution may differ significantly from their fluorescence decay properties when present within lesions.

Here, we investigate the sources of FLIm contrast using RS, well-known for its ability to provide highly specific information about the composition of biological samples. Earlier studies demonstrated that Raman is sensitive to the main constituents of atherosclerotic lesions (13, 14) and thus able to provide extensive compositional information. In this work, the dataset consists of co-registered FLIm and Raman data from *ex vivo* human coronary samples acquired with a combined FLIm and Raman imaging probe. An endmember identification technique is applied to the Raman data to identify the different lesion constituents. Histological evaluation at select locations is used to verify that these findings are consistent with previously reported results. A descriptive modeling approach based on linear regression models quantifies links between FLIm contrast and intensity variations of specific Raman spectral bands, improving on the more qualitative interpretation of multimodal imaging data reported in literature (15–17). This method is used to identify the molecular species associated with fluorescence lifetime variations, a critical step to support the adoption of FLIm as a quantitative tool for the characterization of atherosclerotic lesions biochemical makeup.

## Methods

### FLIm-Raman Instrumentation

The bimodal FLIm-Raman scanning fiber optic probe used to image the arterial specimens was adapted from a previously reported probe configuration, with improved fiber arrangement and distal-end optics (18, 19). Briefly, the probe consists of one central fiber surrounded by nine peripheral fibers, all 300 μm-core UV low-OH fluorine doped fused silica. The central fiber, without distal end filter, is used for FLIm excitation and collection. One of the peripheral fibers is used for Raman excitation (785 nm) and is combined with a low-pass filter to suppress background fiber signal. Another unfiltered peripheral fiber is available for FLIm for separate excitation/collection paths (unused). The seven other fibers, combined with a high-pass filter that rejects the 785 nm excitation light, are collecting the Raman signals. The distal end optic consists of a 2 mm diameter 0.2 NA lithium doped GRIN lens, suitable for use in combination with 355 nm FLIm excitation. The working distance of the probe was determined experimentally at 1–1.5 mm for Raman and FLIm.

The Raman light source consists of a 785 nm multimode laser [0811A100-B model/Ocean optics (Innovative Photonic solutions)]; the output power at the end of the probe was set at 93 mW. The collection fibers were connected to a Raman spectrometer (LS785, Princeton Instruments) with a reflective grating (830 grooves/mm, Princeton Instruments) and equipped with front illuminated CCD open electrode camera (PIXIS-256B Princeton Instruments). Detection was performed using a thermoelectrically cooled detector at −70°C, with 1 s exposure time and 10 accumulations per measurement point. The spectral range was 200–3,500 cm^−1^ with a spectral resolution of about 15 cm^−1^.

FLIm was performed using a pulse sampling setup developed by our group (20). This instrument enables the collection of entire fluorescence decay measurements over four different wavelength channels from each excitation pulse. The excitation was generated using a Nd:YAG microchip Q-switched laser frequency tripled to 355 nm (Teem Photonics, France) with an externally controlled repetition rate of 4 kHz and a pulse energy of ~1.23 μJ. The central wavelength and spectral widths of the instrument channels are determined by a series of dichroic mirrors and bandpass filters and are defined as follows: 387/35 nm (CH1), 443/29 nm (CH2), 546/38 nm (CH3), and 628/53 nm (CH4) and are comparable to spectral bands used for previous studies (6, 19, 21). A temporal multiplexing scheme using different lengths of delay fibers (1/13/25/37 m) is used so that the contributions from each channel are delayed in time (60 ns increment), thus enabling the use of a single photodetector (Microchannel plate photomultiplier tube, R3809U-50, Hamamatsu, JP), amplifier (AM-1607-3000, Miteq Inc., USA) and high-speed digitizer (12.5 GS/s, 3 GHz, PXIe-5185, National Instruments, TX). To improve signal to noise ratio, 64 waveforms were acquired and averaged for each sample location.

### Data Acquisition

The bimodal fiber probe enables the simultaneous acquisition of FLIm and Raman data, but their respective acquisition speeds are very different (0.016 s/point for FLIm, 10 s/point for Raman). Therefore, the imaging sequence was implemented as follows. The combined FLIm–Raman probe was mounted on a 3D X-Y-Z translational stage (PROmech LP28, Parker, Charlotte, NC) used to scan the sample in a grid pattern with a step size of 250 μm × 250 μm. A raster scan of the whole sample was performed with FLIm (typical duration: 1–2 min). Based on reconstructed 2D FLIm images, 19 regions of interest (ROI) characterized by various by lifetime levels in CH1, CH2, and CH3 of the instrument were selected for interrogation using RS. Raman data acquisition was performed using the same 250 μm × 250 μm spatial sampling.

### Sample Preparation, Imaging, Histology

Six human coronary artery samples were obtained from the University of Pennsylvania heart transplant in compliance with current legal requirements and guidelines and was under approval by the University of Pennsylvania Hospital Institutional Review Board as well as UC Davis Biological Use Authorization. Prospective informed consent for research use of heart tissue was obtained from all transplant recipients and next-of-kin in the case of organ donors. Coronary artery samples were harvested from hearts, chilled in isopentane, frozen in liquid nitrogen and stored at −80°C. Before imaging, each sample was thawed, and dissected longitudinally to expose the lumen. The samples were then sutured on a calcium fluoride slide to flatten the lumen surface and prevent tissue motion. Imaging was performed at room temperature and samples were kept hydrated using phosphate buffer saline solution. The entire surface of samples was imaged using FLIm, and 7,291 Raman spectra were collected from the 19 ROIs.

After imaging, the specimens still mounted on the holders were fixed in 10% buffered formalin. Histology processing was performed at the Texas Heart Institute (Houston, TX). Samples were first imaged using high resolution planar X-ray (Faxitron MX-20, Lincolnshire, IL). Samples were then decalcified, sliced in locations corresponding to FLIm-Raman scans, paraffin embedded, and sectioned (Supplemental Figures 1, 2). Staining using hematoxylin and eosin (H&E) and Movat's pentachrome were performed to visualize plaque constituents. Immunohistochemical staining with CD68 was performed to visualize the presence of macrophage foam cells (mFC). Registration of imaging data with histology is performed based on sample outline and gross sectioning locations (Supplemental Figures 1, 2).

### Data Analysis

FLIm data were processed using a method previously reported by our group that enables fast and robust estimation of fluorescence decay parameters technique (22). Briefly, the fiber probe background was subtracted from the acquired waveform, and the contribution from each channel was separated and processed independently. A constrained least-square expansion technique using a set of Laguerre basis functions was used to obtain tissue fluorescence decays, from which average lifetimes were extracted for each point measurement and each channel of the instrument. En face FLIm images were reconstructed based on raster scanning parameters. CH4 (628 nm) signal was characterized by a low signal to noise ratio (mean SNR = 20.6 dB) due to weak fluorescence emission in that spectral range and was therefore not included in the analysis.

Histology registration with imaging data was performed based on gross sectioning location. In several sections, calcification visible in histological sections (L2/L4/L5/L9), as well as planar X-rays of the samples, enabled further confirmation of the histology sections location with respect to spectroscopic imaging data.

**Figure 1:**
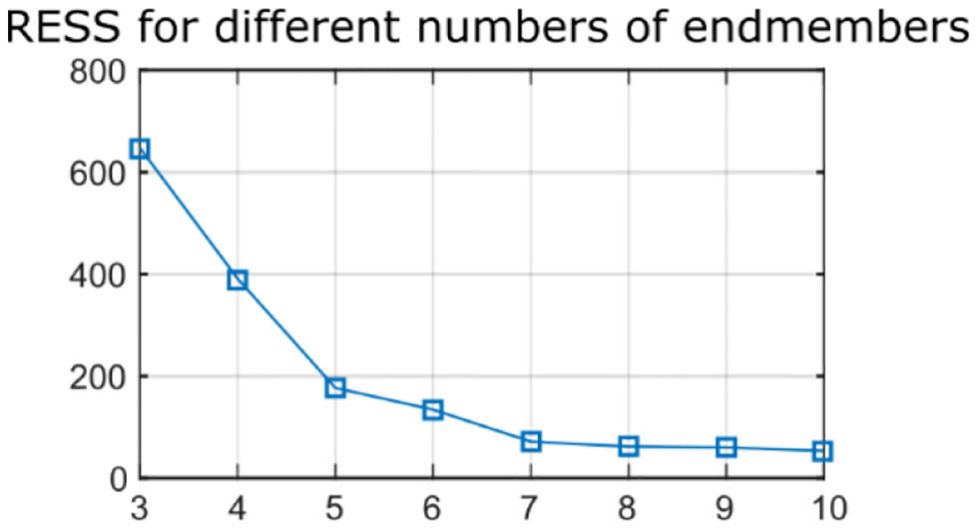
Residual error sum of squares (RESS) for Raman data reconstructed with increasing number of endmembers (*n* = 3–10). Seven endmembers enable accurate reconstruction of the data.

Raman signal recorded from the samples presented an autofluorescence background as well as etaloning artifacts from the CCD. Raman data were processed as follows. A wavenumber calibration was performed based on spectra from a reference sample (paracetamol) (23). A baseline correction to remove the autofluorescence contribution was performed using a sensitive non-linear iterative peak algorithm (SNIP) (24). Spectra were truncated to the 650–1,800 cm^−1^ wavenumber range and a vector normalization was applied. Etaloning artifacts were removed by reconstructing the spectra from the subset of first 10 artifact-free principal components. Finally, an endmember decomposition was performed with various numbers of endmembers (*n* = 3–10) using the unmixR package in R (25). The residual error sum of square (RESS) was computed, for an increasing number of endmembers, by reconstructing all acquired spectra as a linear combination of the endmembers and comparing the reconstructed spectra with the original data. Increasing the number of endmembers up to seven leads to a large reduction in the RESS, whereas additional endmembers beyond seven do not provide a noticeable reduction in RESS, therefore a number of 7 endmembers was retained for the rest of the study (Figure 1). In addition to the endmember determination based on a qualitative assessment of RESS improvement, it was observed that an 8-endmember decomposition led to two endmembers with very similar spectral shapes, and thus did not enable the identification of additional tissue components.

For each measurement point of the Raman ROIs, a high number of parameters were available (average lifetime for each FLIm spectral band, intensity for each Raman wavenumber). The relationship between variations of fluorescence lifetime values for each spectral channel and variations of Raman spectral features was investigated using linear regression analysis (26). It is expected that RS provides more information about the samples, and in order to investigate which species are likely to generate FLIm contrast, a regression model predicting Raman intensity based on FLIm information was computed.

**Figure 2:**
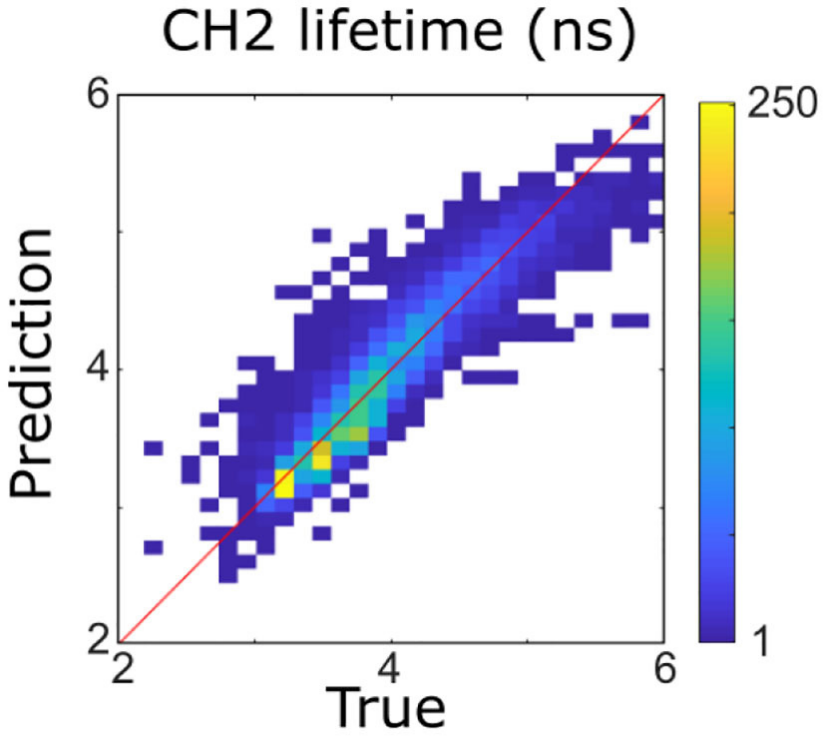
Four hundred forty-three nanometers lifetime was accurately predicted (*R*^2^ = 0.89) from 387 to 546 nm lifetimes using a multivariable linear model. It was therefore not considered for the rest of the analysis.

As a first step, multicollinearity of FLIm lifetimes across the different wavebands was evaluated by computing the coefficient of multiple determination obtained by regressing the lifetime of each FLIm spectral band on the lifetimes of the other two channels. The highest value was obtained for CH2 (*R*^2^ = 0.89), so this band was excluded from the analysis (Figure 2). The coefficient of multiple determination computed between the *LT1* (CH1 lifetime) and *LT3* (CH3 lifetime) was low (*R*^2^ = 0.16) so collinearity between lifetime parameters derived from these two spectral bands was not an issue and interpretation of the regression vectors was meaningful (27).

The relationship between the FLIm and Raman datasets was investigated by applying a multivariate multiple linear regression model (26), using *LT1* and *LT3* as independent variables, and the Raman intensities for each wavenumber *y* as dependent variable, per Equation (1):

(1) $$\begin{array}{l} y_{ik}=b_{0k}+b_{1k}LT1+b_{2k}LT3+\varepsilon_{ik} \end{array}$$

Where *i* is one of the 7,291 measurements, *k* is one of the 428 wavenumbers of the Raman spectra in the fingerprint region, and ε is an error term.

The regression model can be expressed in matrix form:

(2) $$\begin{array}{l} Y=X\beta +\varepsilon \end{array}$$

*Y* is the 7,291 × 428 response matrix and consists of the Raman intensity for each measured location and wavenumber. *X* is the design matrix and consists of a column of ones and the *LT1* and *LT3* for each measured location. β is the 3 × 428 matrix of coefficients where the first row is the *y*-intercept and the 2nd and 3rd rows correspond to the expected changes in Raman spectra per unit of change in *LT1* and *LT3*, respectively. For simplification, the regression was performed on the centered FLIm variables. In that case, the *y*-intercept (offset) is the mean of the data. A cross validation scheme where each ROI was left out of the analysis was used. The matrix of coefficients $\hat{\beta }$ was estimated using an ordinary multivariate normal maximum likelihood estimation using Matlab (2019a, Mathworks, Natick, MA) excluding, in turn, data from a single ROI. The regression model was then used to predict Raman intensities from that ROI (**Figure 6**). Mean and standard deviations of the regression vectors computed for each cross validation were computed (**Figure 6**).

The Raman bands that correlate most with lifetime parameters were determined by computing the fitted Raman spectra:

(3) $$\begin{array}{l} \hat{Y}=X\hat{\beta } \end{array}$$

Where Ŷ = (ŷ_*i* 1_, …ŷ_*ik*_, …, ŷ_*i* 428_) correspond to the predicted Raman intensities for each point measurement.

The coefficient of multiple determination ${\text{R}}_{\text{k}}^{2}$, which corresponds to the ratios of regression sum of square divided by the total sum of squares, is computed for each wavenumber *k* according to Equation (4):

(4) $$\begin{array}{l} {R_{k}}^{2}=\frac{\sum_{i=1}^{n}{\left(\hat{y_{ik}}-\bar{y_{k}}\right)}^{2}}{\sum_{i=1}^{n}{\left(y_{ik}-\bar{y_{k}}\right)}^{2}} \end{array}$$

Where *n* is the number of point measurements, and *y*_*k*_ is the average of the measured Raman intensities for the *k*th wavenumber.

The coefficient of multiple determination, represented as a function of wavenumber, allowed the identification of regions of the spectra that demonstrated the highest correlations with variations in fluorescence lifetime (**Figure 6**).

To better identify which Raman spectral band correlated the most each lifetime parameter, a single parameter prediction based on the model identified above was performed, using in turn only one measured lifetime parameter while fixing the other lifetime parameter. *LT1* prediction was assessed by replacing the observed *LT3* values in the design matrix by their average value and computing the corresponding fitted Raman spectra and coefficient of determinations using (Equations 3, 4). LT3 prediction was assessed by computing the coefficient of determination when replacing the *LT1* values in the design matrix by an average value (**Figure 6**).

Finally, the measurement points were partitioned in four subsets based on *LT1* and *LT3* values (above or below 4 ns), and the average Raman spectra of each subset were plotted to confirm the differences identified using the method described above (**Figure 7**).

## Results

### FLIm

**Figure 3:**
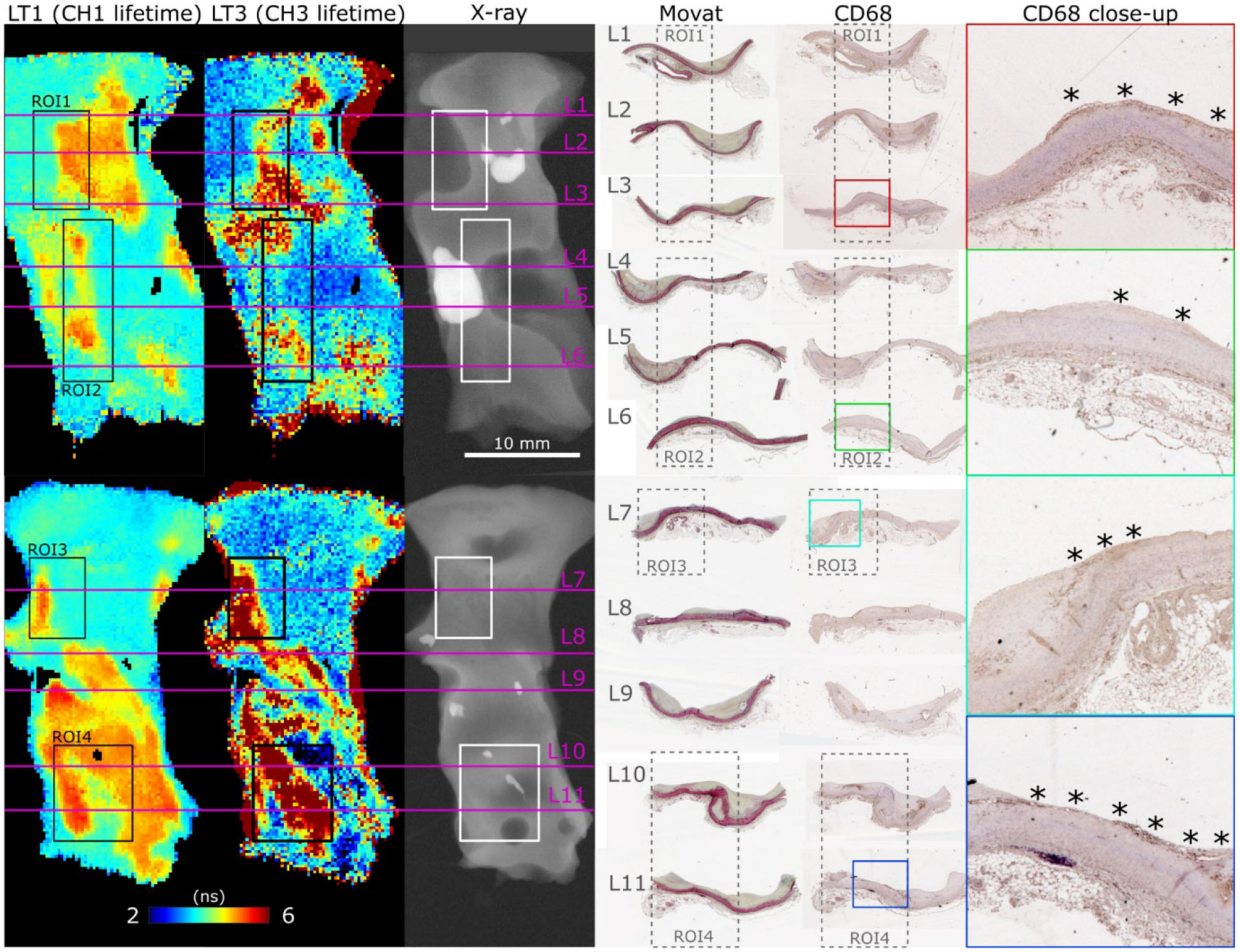
En face FLIm maps of LT1 (387/35 nm lifetime) and LT3 (546/38 nm lifetime) for two samples illustrate the strong lifetime variations in both channels. ROI 1–4 correspond to locations where both FLIm and Raman data were acquired. Planar X-ray highlights calcification location. Histology sections (L1–L11) were positioned based on gross sectioning location and presence of calcification. Increase of LT1 was always observed in combination with intimal thickening, but some locations presented a thick intima without corresponding increase in lifetime (L4, L5, left side of ROI2). The source of LT1 was investigated in combination with Raman spectroscopy in the next section. LT3 corresponded to presence of superficial mFC, visible from the CD68 stained sections (marked with *).

FLIm 2D maps of LT1 and LT3 illustrate the strong contrast observed from different regions of diseased vessel. Representative results from two samples are depicted in Figure 3, and more detailed presentation of histological findings is available in the Supplemental Section. Movat's pentachrome stained sections highlight that LT1 was only increasing in presence of intimal thickening. However, we observed that some locations characterized by a thickened intima do not present increased LT1 (e.g., the fibrocalcific lesions on the left side of ROI2 corresponding to sections L4 and L5). Many molecular species (e.g., some lipids, collagen, and elastin) present emission in the near-UV/blue regions (6, 10) and may cause the observed LT1 variations. The increase of LT3 was associated to the presence of mFCs as confirmed by CD68 stained sections. Another contributor of increased LT3 was perivascular adipose tissue. The penetration depth of FLIm excitation is limited, therefore perivascular adipocytes are not visible through the intima, but the adipocytes signature can be readily identified at the edges of the samples.

### Raman Spectroscopy

**Figure 4:**
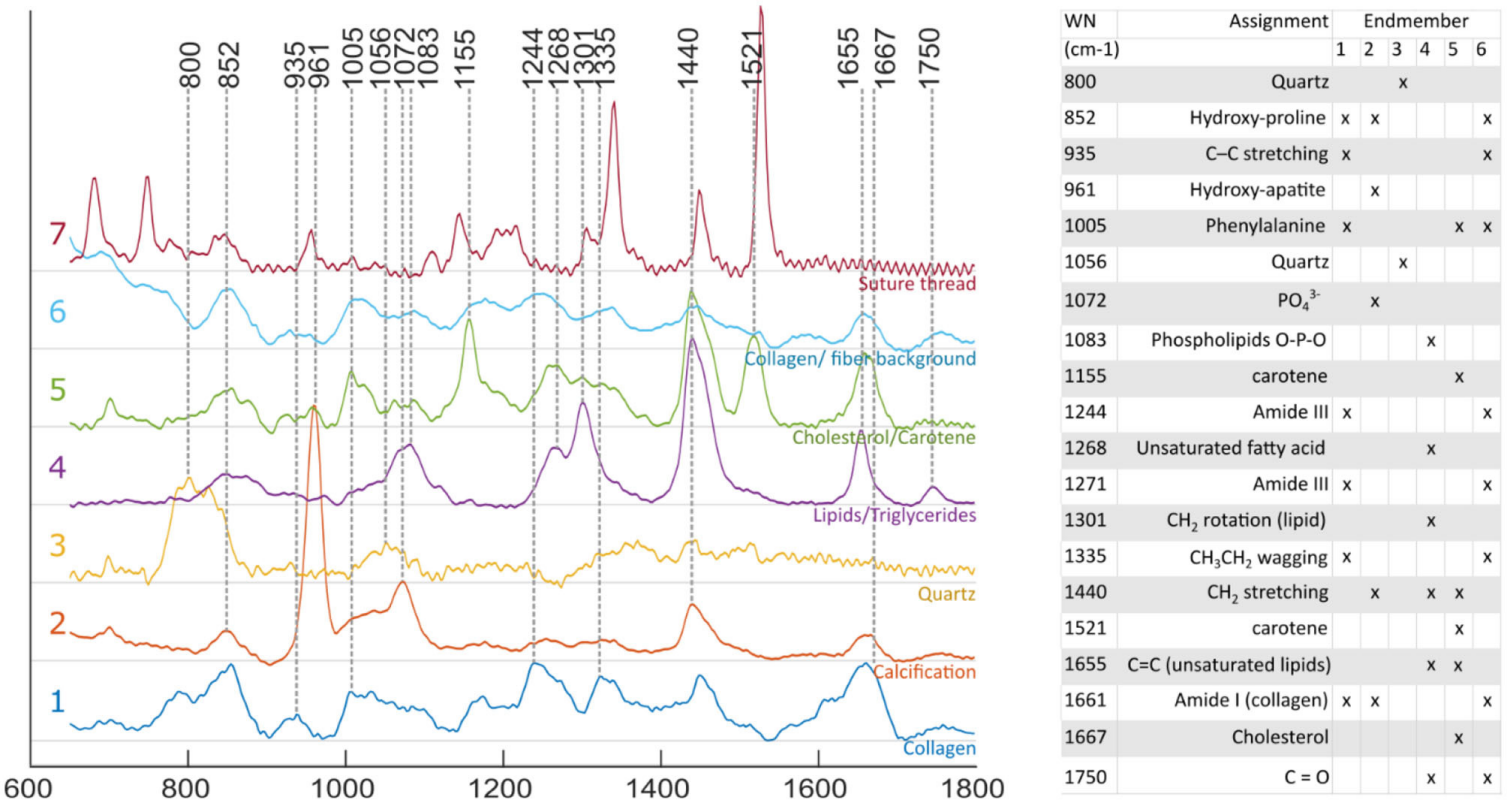
Raman spectra of endmembers. Endmember decomposition was consistent with species expected from atherosclerotic lesions, and other elements present in the field of view [1, collagen; 2, calcification; 3, quartz (probe: fiber optic, distal lens); 4, lipid rich (unsaturated triglycerides); 5, cholesterol/ carotene rich; 6, collagen/ fiber background; and 7, suture thread].

Raman spectra obtained for individual locations were affected by noise despite the extended exposure time, limiting the ability to identify spectral features from single spectral measurements. Using the endmember technique reported here, it was possible to mitigate this limitation by expressing each measurement as a linear combination of the endmembers, and therefore extract robust compositional information at each location. Identification of species contributing to each endmember was performed based on known emission bands (16, 28, 29) of species expected in atherosclerotic lesions, combined with spatial information. Four endmembers relate to the actual lesion: collagen, calcification, triglycerides and cholesterol/carotene (Figure 4).

**Figure 5:**
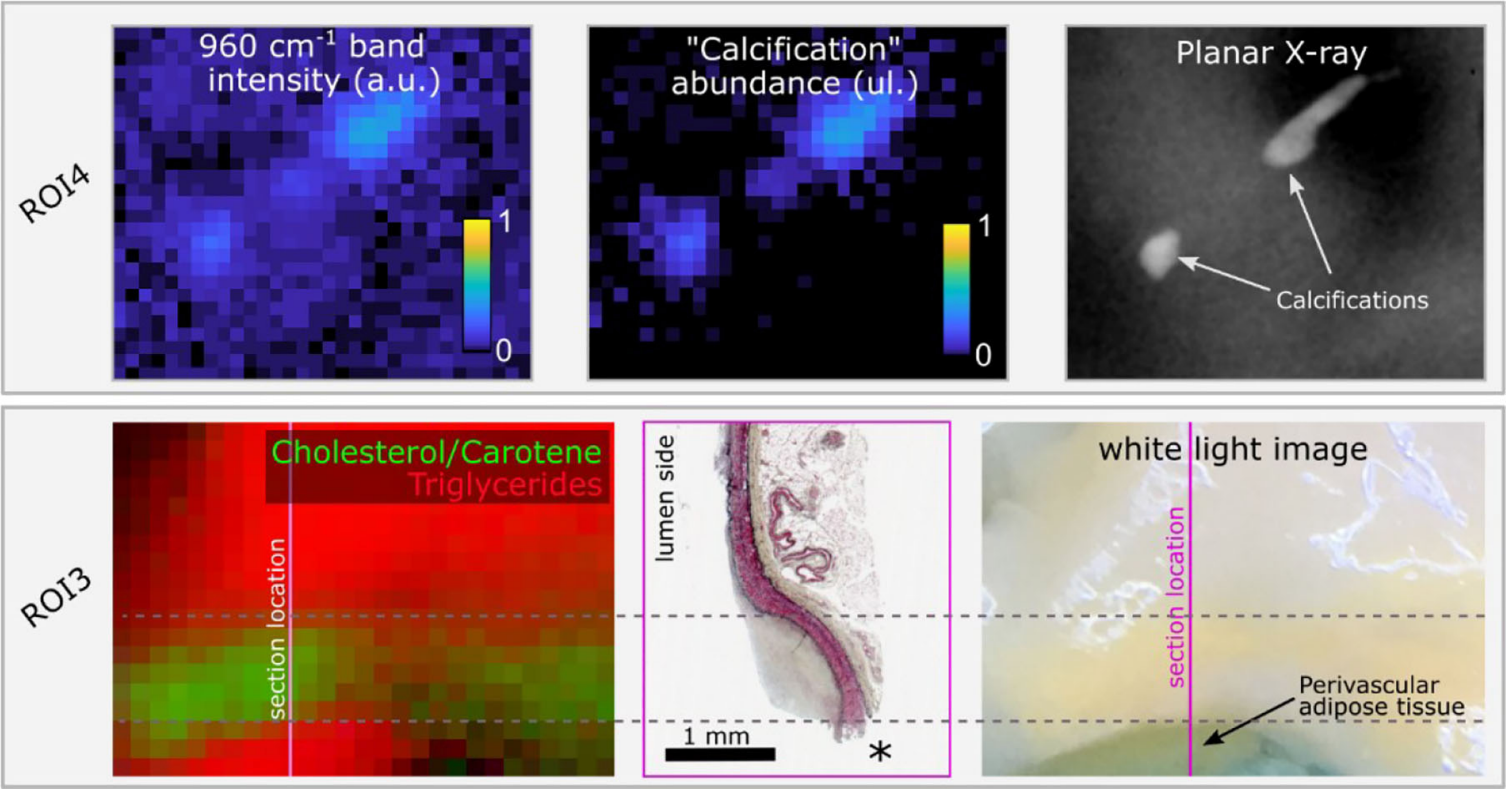
Raman hydroxyapatite band intensity map (960 cm^−1^) compared with “Calcification” endmember abundance map for ROI4 and ground truth location of calcifications obtained from planar X-ray. The two small calcifications are easily visible with both methods. The endmember image correctly shows no signature from calcifications over the rest of the sample, whereas the intensity image shows elevated background (upper panel). In ROI3, the color image represents the abundance of endmembers 4 (“triglycerides,” red) and 5 (“cholesterol/carotene,” green). Two distinct locations present an abundance of the “triglycerides” endmember. The lower spot corresponds to perivascular adipose tissue, rich in triglycerides, exposed at the edge of the sample, easily identified in the white light image, and lost during histology processing (location marked as *). The upper area corresponds to a diffuse intimal thickening region where perivascular adipose tissue contributes to the Raman signature due to the reduced wall thickness. In between, the region of predominant cholesterol/Carotene signature overlaps with the lesion location (lower panel).

The advantage of the endmember identification technique is illustrated by the comparison of direct mapping of the 960 cm^−1^ hydroxyapatite peak intensity compared with mapping of the “calcification” endmember abundance (Figure 5) for a region that presents two small calcification areas. The endmember map demonstrated better quantification with negligible contribution of calcification over the rest of the field of view, whereas the band intensity image presented an elevated background. Mapping of the locations of triglycerides and Cholesterol/Carotene endmembers highlights the differences in their spatial distribution (Figure 5).

### Combined FLIm-Raman

**Figure 6:**
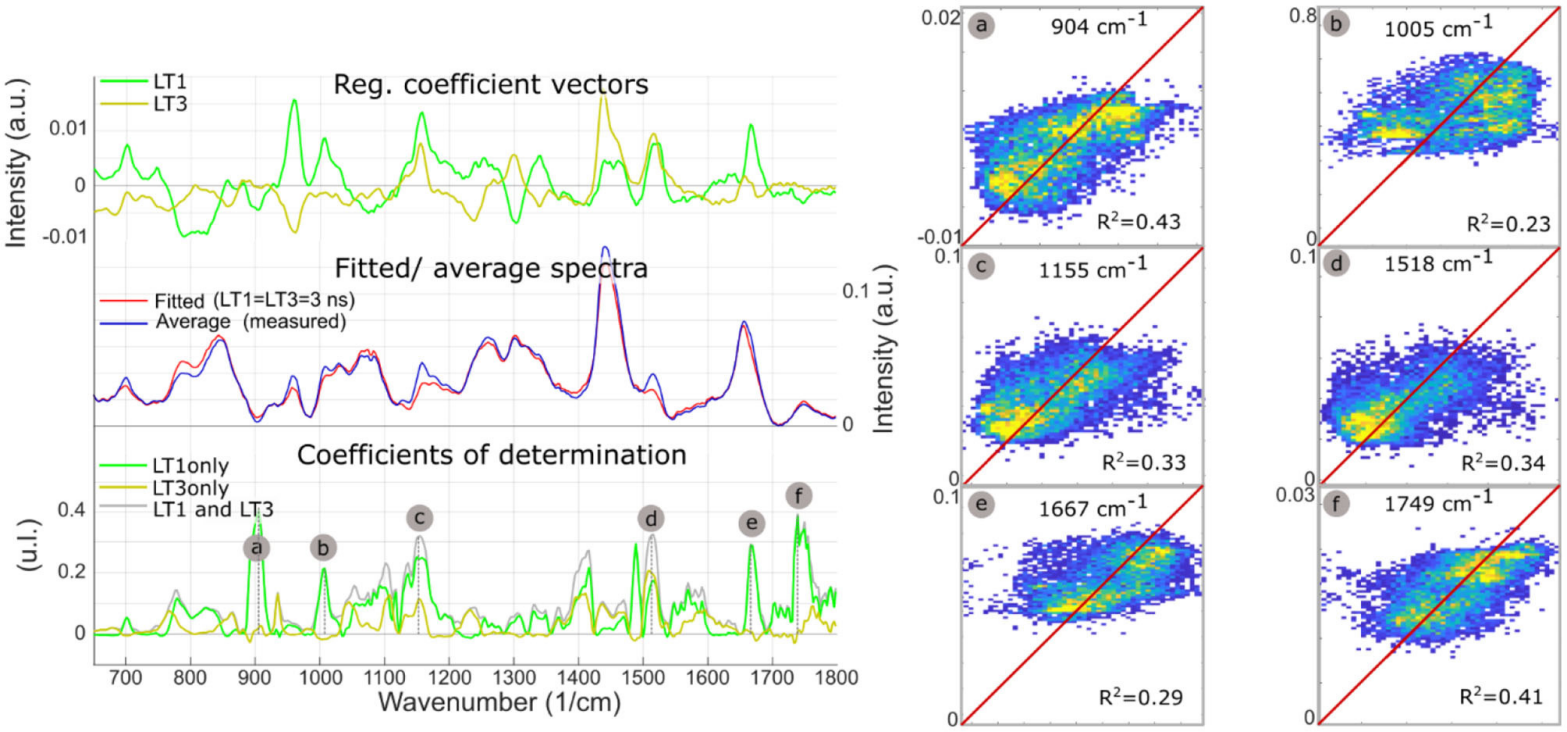
LT1 and LT3 regression coefficient vectors are expected variations of Raman spectra for a respective unit increases of LT1 and LT3. The standard deviation of the regression vectors for each cross-validation subset is represented by the shaded area behind each curve. The offset vector of the regression is equal to the average Raman spectrum. Averaged measured Raman spectrum and modeled spectrum for diffuse intimal thickening regions (LT1 = 3 ns, LT3 = 3 ns), present few differences. Plot of coefficients of determination between measured Raman intensities and Raman intensities predicted from LT1 and LT3 (gray) demonstrates that the amount of variance that can be explained by lifetime variations varies for the different regions of the spectra: as expected, not all Raman bands can be predicted from FLIm values. Some locations show a correlation coefficient >0.3 (a, Protein/ Collagen; b, Phenylalanine/ Carotene; c/d, Carotene; e, Cholesterol; f, Ester bond). Prediction using lifetime from only one channel of the instrument (and replacing the other lifetime information by the average for the whole dataset) highlights which lifetime channel contributes most to the prediction (LT1: a/b/c/e/f). The predicted vs. measured intensities are represented for bands a/b/c/d/e/f (right panel).

Figure 6 summarizes the findings from the multiple regression analysis. The regression coefficient vectors represented expected variations of Raman intensities from the average spectrum, for respective unitary increases of LT1 and LT3. Low values for a given wavenumber mean that the variance of the Raman data for this wavenumber was low, or that the FLIm values did not explain variations in Raman intensity. Conversely, high values of regression coefficients may only derive from high variance in the Raman data. The amount of Raman intensity variance explained by variations in fluorescence lifetime was represented by the coefficient of determination vector. Some of the Raman bands that stood out in the *R*^2^ plot corresponded to higher values in the regression vectors as well, such as bands “b/c/d/e.” Conversely, the 960 cm^−1^ band, linked with hydroxyapatite, presented high regression coefficient values but an *R*^2^ close to zero, highlighting that observation of the regression coefficients was not sufficient to evaluate the strength of the relationships between datasets. On the contrary, band “a” had the highest *R*^2^, and low regression coefficient values, explained by the low Raman intensity observed in this band.

The single parameter prediction highlighted that LT1 primarily explained Raman intensity changes, except for band “d” (carotene), equally split between LT1 and LT3. LT1 increase was associated with increases in Raman bands associated with carotene and cholesterol, pointing to LDL accumulation as the origin of LT1 FLIm contrast. The single fluorescence channel prediction described here is not equivalent to a linear regression using LT1 or LT3 as unique independent variable that would be subject to omitted-variable bias: a simple regression is affected by the correlation between LT1 and LT3 caused by the colocalization of different species in atherosclerotic lesions.

Plots of measured vs. predicted Raman intensities (Figure 6, right panel) illustrate the predictive value of the regression model. Raman intensities in specific bands were impacted by variations in baseline subtraction between different measurements as well as the vector normalization scheme applied here, where increased emission in one band led to a relative reduction of the other bands. Because none of these effects were modeled by FLIm, the residual variance was expected to remain high even if the underlying relationships between Raman and FLIm signature were properly identified.

**Figure 7:**
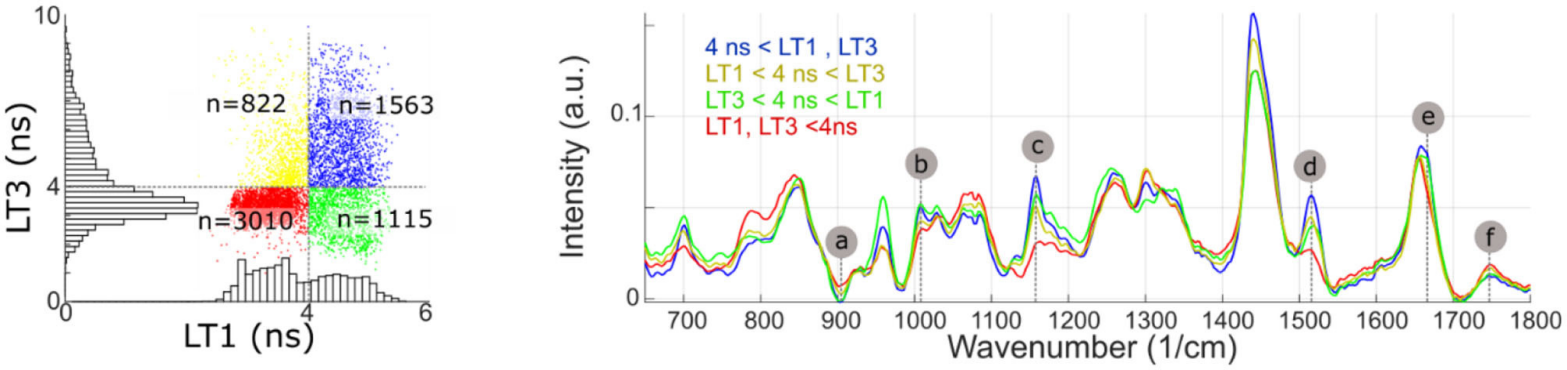
Measurement points are partitioned based on LT1 and LT3 values (above or below 4 ns). The average Raman spectra of each subset present noticeable differences in bands identified from the regression analysis (a, Protein/ Collagen; b, Phenylalanine/ Carotene; c/d, Carotene; e, Cholesterol; f, Ester bond). Some of the regions where average spectra present noticeable differences (785, 960, and 1,440 cm^−1^), conversely, only present very weak correlation with fluorescence lifetime.

Identification of the Raman bands intensity variations highly correlated with fluorescence emission properties, using the regression method described above, were confirmed by subdividing the imaging dataset based on LT1 and LT3 values (threshold: 4 ns for both bands). The findings, presented in Figure 7, confirmed the validity of the approach. Average Raman spectra for each subset presented differences in correspondence to bands a-f identified from the regression analysis.

## Discussion

The current study demonstrated that the molecular specificity of RS allowed for the identification of molecular species and/or composite of species resulting in changes in FLIm parameters in distinct spectral bands. More specifically, fluorescence lifetime increase in the violet spectral band was linked with increase in cholesterol and carotene Raman band intensities, indicative of LDL accumulation. These findings derive from a descriptive modeling approach (30) that was for the first time adapted to the analysis of FLIm-Raman imaging data.

While the autofluorescence spectra of biological compounds found in arterial vessels have distinct spectral peak emission, their emission is typically broad. Thus, each of the four spectral channels of the FLIm system is expected to capture varying amounts of signal from distinct sets of fluorophores. The first channel (violet spectral band) presented a significant lifetime increased for areas associated with atherosclerotic plaques. Earlier studies have associated the emission in this spectral band to that of structural proteins and mainly collagen in advanced lesions (31). However, measurements of pure compounds excited by 355 nm indicate that both structural proteins as well as lipids present violet autofluorescence emission (6), therefore other species such as lipids or proteoglycans could contribute to the lifetime increase observed in the violet channel. The histological analysis of coronary specimens has indicated the presence of collagen, proteoglycans as well as extracellular lipids, thus the direct identification of the molecular specie responsible for the observed LT1 increase was not possible. The FLIm results for the third emission channel (green spectral band) were found consistent with previous reports that linked lifetime increase in this band with mFCs accumulation (6, 32). The results for the second emission channel (blue spectral band) show that for these samples, the computed lifetime is to a large extent a linear combination of the violet and green bands, leading to its exclusion from the analysis.

Analysis of Raman using an endmember extraction, in combination with histology sections, yielded results consistent with previously reported work (13, 14). The regions presenting the most triglyceride contributions are areas where perivascular adipose tissue is exposed at the edge of the sample, but also regions of minimal intimal thickening. In that latter case, the total wall thickness of ~200 μm and the large Raman cross section of lipids compared to proteins explain why the Raman signature is dominated by contributions from tissue located outside of the vessel. This association of triglycerides with perivascular adipose tissue was previously reported in the literature (13, 16). The second lipids contribution, related to cholesterol/carotene, was expressed in areas of increased intimal thickness, in agreement with earlier studies (16). Carotene presents a strong Raman cross section due to pre-resonance enhancement and is readily embedded in LDL, therefore its colocalization with cholesterol was expected. Cell studies on the tracking of LDL uptake into macrophages have taken advantage of this cholesterol/β-carotene colocalization (33). Applied in the context of biological human samples, the same cholesterol/β-carotene association highlighted LDL accumulation into the arterial wall.

The analysis of the regression model linking Raman and FLIm measurements identified a relationship between LT1 increase and accumulation of carotene and cholesterol associated with LDL accumulation in developing lesions, as well as decreased contribution from perivascular adipose tissue triglycerides, due to increased intimal thickness. Perivascular adipose tissue is beyond the penetration depth of FLIm so the main contributing factor to variations in LT1 was attributed to LDL accumulation. On the other hand, collagen signature is readily identified in the Raman data but no correlation with variations of FLIm signature was observed. As a consequence, previous FLIm studies of atherosclerosis samples corroborated with histology that had associated lifetime contrast in that wavelength band with variations in collagen content may need revisiting (13, 34). These earlier findings may have stemmed from the ubiquitous presence of collagen in atherosclerotic lesions and the difficulties in properly characterizing lipids contribution in histology studies. This new finding highlights the superior ability of RS to perform direct, quantitative, lipid profiling. Variations of LT3 presented only weak relationships with Raman features. LT3 increase derives mostly from bright ceroids autofluorescence (34, 35), present in small amount and not known to present a high Raman cross section.

The method used to identify sources of FLIm contrast is constrained by the ability of RS to detect specific molecular species. For example, the contribution to the FLIm signature of species not identified in the Raman signal, such as proteoglycans, cannot be assessed with this approach. Proteoglycans are typically co-localized with LDL accumulation (36), thus the findings reported in this study cannot exclude that the contrast observed in LT1 is caused by proteoglycan accumulation. The performance of the regression model is also limited by the characteristics of the Raman data. Raman variance is explained by variations in tissue composition, but also variations in baseline correction, influence of vector normalization, and detection noise. Therefore, values of coefficients of multiple determination (*R*^2^) observed for the regression model (<0.4) are expected, and adequate to support the findings of this descriptive study. Another confounding factor for this regression analysis is the difference in interrogated volume between FLIm and Raman. Here, 355 nm FLIm excitation is characterized by a low penetration depth in tissue, whereas 785 nm Raman excitation readily propagates in tissue up to 400–600 μm. Therefore, the contribution to the Raman signal of triglyceride in perivascular adipose tissue prevented the identification of possible triglycerides contribution within the FLIm penetration depth.

The availability of co-registered FLIm and Raman data enabled a model-based approach to the identification of relationships between FLIm and Raman variations. This approach, called descriptive modeling, is well-suited to the systematic and quantitative analysis of data consisting of a large number of variables, as is the case here. This modeling approach differs from previous comparative imaging studies reporting the combination of Raman imaging with other optical imaging modalities such as near-infrared spectroscopy, coherent anti-Stokes RS, or Fourier transform infrared spectroscopy (15–17) where the ability of each modality to identify specific components in the imaged sample was evaluated qualitatively or quantified on aggregated locations. A model-based approach, however, requires spatially co-registered datasets from both imaging modalities. Earlier comparative imaging studies using RS consisted of datasets acquired with dedicated (independent) instrumentation that differed in field of view, resolution, and/or spatial sampling, and thus were not suitable for a model-based approach. In contrast, the model-based approach described here enabled systematic and quantitative assessment of the presence of specific components. A limitation in the reported implementation is the mismatch of penetration depths of each modality, that could be addressed by matching the penetration depth of RS with the penetration depth of FLIm using a confocal probe design.

Predictive modeling approaches based on RS in combination with other analytical methods have been reported for a wide variety of applications, such as cell (37) or transcriptome (38) identification, or active pharmaceutical ingredient content quantification (39). Both predictive and descriptive modeling may rely on the same tools (e.g., multiple regression techniques), but the purpose of predictive approaches is to use high dimensional Raman data to develop non-invasive prediction models of specific phenotypes or components. The emphasis was therefore on the performance of the classifier. However, the exact identification of the Raman spectral features that allowed differentiation, a key finding of this study, was not evaluated.

In this study, FLIm and Raman modalities were integrated in a single optical fiber imaging probe. This approach presents clear advantages for accurate registration of imaging datasets, but at the expense of the optical performance for each modality. While the compact fiber optic probe design is ideally suited to contact measurements on a variety of samples, when used to image irregularly shaped samples such design leads to variations in the probe to tissue distance across the field of view. For this dataset, a percentile ratio (90/10) of ~3.3 was observed for the Raman intensity, meaning that signal collection was suboptimal in many locations. This could be addressed by performing an imaging scan where the imaging probe follows a 3D trajectory and maintains a consistent distance with tissue. Alternatively, an inverted setup, where imaging is performed through a transparent window and thus the sample to probe distance can be accurately maintained could be used (40). Ultimately, a setup able to perform FLIm-Raman imaging on thin (<100 μm) tissue sections would also present the benefit of reducing the effect of differences in penetration depths between modalities and therefore represent an ideal tool for future FLIm/Raman comparative imaging, at the expense of additional sample preparation.

Validation of a new spectroscopic imaging technique by means of co-registration with an established reference modality (e.g., Raman spectroscopy characterized by high molecular specificity) requires reduced human intervention compared to histology-based validation. Sample preparation is minimal and data analysis requires little human input beyond the initial definition of the methods. Thus, expansion to a much higher sample number can be achieved more readily than with histopathology-based validation studies where human operations in sample processing, registration, and feature extraction is a bottleneck.

## Conclusion

The ability of FLIm to provide label-free molecular contrast with a device suitable for the acquisition of data *in vivo* shows potential for improved characterization of atherosclerotic lesions, but identification of specific species based on FLIm contrast alone is a challenge due to the variety of endogenous fluorophores present in diseased arteries. On the other hand, several technical challenges limit the potential of Raman spectroscopy for *in vivo* interrogation of the vasculature, but its high specificity enables the identification of key components of atherosclerotic lesions. By combining both approaches, we identified in *ex vivo* human samples that the source of fluorescence lifetime increase in the violet spectral range observed in atherosclerotic lesions, previously thought to be linked with increased collagen content, is associated with LDL accumulation. This finding illustrates that histology-based validation of imaging techniques may be complemented by a multimodal imaging approach combined with a model-based data analysis, where a high specificity spectroscopic imaging method (Raman) is used to shed light on a high speed/lower specificity modality (FLIm). A broader range of imaging validation studies may benefit from such approach, due to the ability to detect molecular species not readily identified in formalin fixed/paraffin embedded histological sections, and the availability of data analysis methods suited to the efficient analysis of co-registered multimodal imaging data. This approach is also easily scalable to the analysis of large numbers of samples as limited user input is required beyond data acquisition, a clear benefit compared to histology-based validation that relies heavily on human operations for sample preparation and feature extraction.

## Data Availability Statement

The datasets generated for this study are available on request to the corresponding author.

## Ethics Statement

The studies involving human participants were reviewed and approved by University of Pennsylvania Hospital Institutional Review Board UC Davis Biological Use Authorization. The patients/participants provided their written informed consent to participate in this study.

## Author Contributions

JB proposed the methodology, performed analysis, and wrote the manuscript. TS developed the Raman data acquisition code and supported data analysis. KM provided tissues for the analysis. JB and TS acquired the multimodal imaging data. TB performed Raman data pre-processing and provided guidance for comparative data analysis. CK assisted in determining chemical species based on Raman information. TB, AA-G, and CK provided input regarding data analysis methods and study findings. TS, TB, AA-G, CK, KM, JP, and LM provided inputs on the revision of the manuscripts. All authors contributed to the article and approved the submitted version.

## Conflict of Interest

The authors declare that the research was conducted in the absence of any commercial or financial relationships that could be construed as a potential conflict of interest.
