# Supplementary material for: Investigating Origins of FLIm Contrast in Atherosclerotic Lesions Using Combined FLIm-Raman Spectroscopy
Source: Front Cardiovasc Med. 2020 Jul 21;7:122. doi: 10.3389/fcvm.2020.00122 (PMC7385056; doi:10.3389/fcvm.2020.00122)
Supplement: Supplementary file 1 [file Data_Sheet_1.docx]

Investigating origins of FLIm contrast in atherosclerotic lesions using combined FLIm-Raman Spectroscopy

Julien Bec^1,2,3*^, Tanveer Ahmed Shaik^3^, Christoph Krafft^3^, Thomas W. Bocklitz^2,3^, Alba Alfonso-Garcia^1^, Kenneth B. Margulies^4^, Jürgen Popp^2,3^, Laura Marcu^1^

^1^Department of Biomedical Engineering, University of California, Davis, California, USA

^2^Institute of Physical Chemistry and Abbe Center of Photonics (IPC), Friedrich-Schiller-University, Jena, Germany

^3^Leibniz Institute of Photonic Technology, D-07745 Jena, Germany

^4^Cardiovascular Institute, Perelman School of Medicine, University of Pennsylvania,

Philadelphia, PA, USA

**SUPPLEMENTAL FIGURES**

**
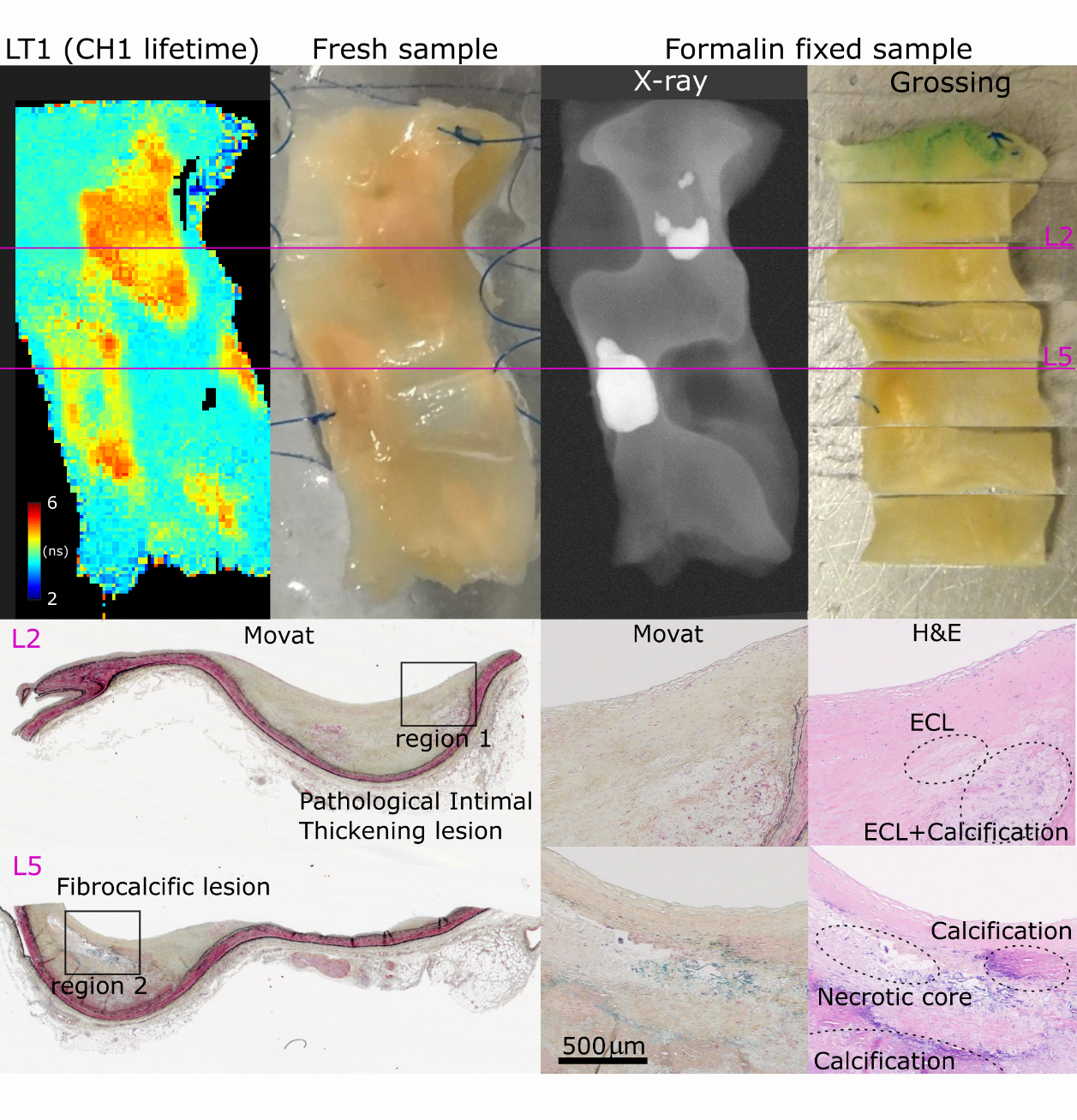
**

**Supplemental Figure 1: Overview of the imaging and histology processing. The sample outline, visible in the en face FLIm image and corresponding white light image of the fresh tissue, enables accurate registration of the FLIm data with the sample. The X-ray image of the sample following formalin fixation highlights that deformations were not observed due to the fixation process. The grossing view enables registration of the stained sections with respect to the imaging data. Detailed histology for sections L2 and L5 highlights healthy areas as well as atherosclerotic lesions characterized by the presence of extracellular lipids (ECL) as well as calcifications (region 1: pathological intimal thickening), and calcifications as well as necrotic material (region 2: fibrocalcific lesion).**

**
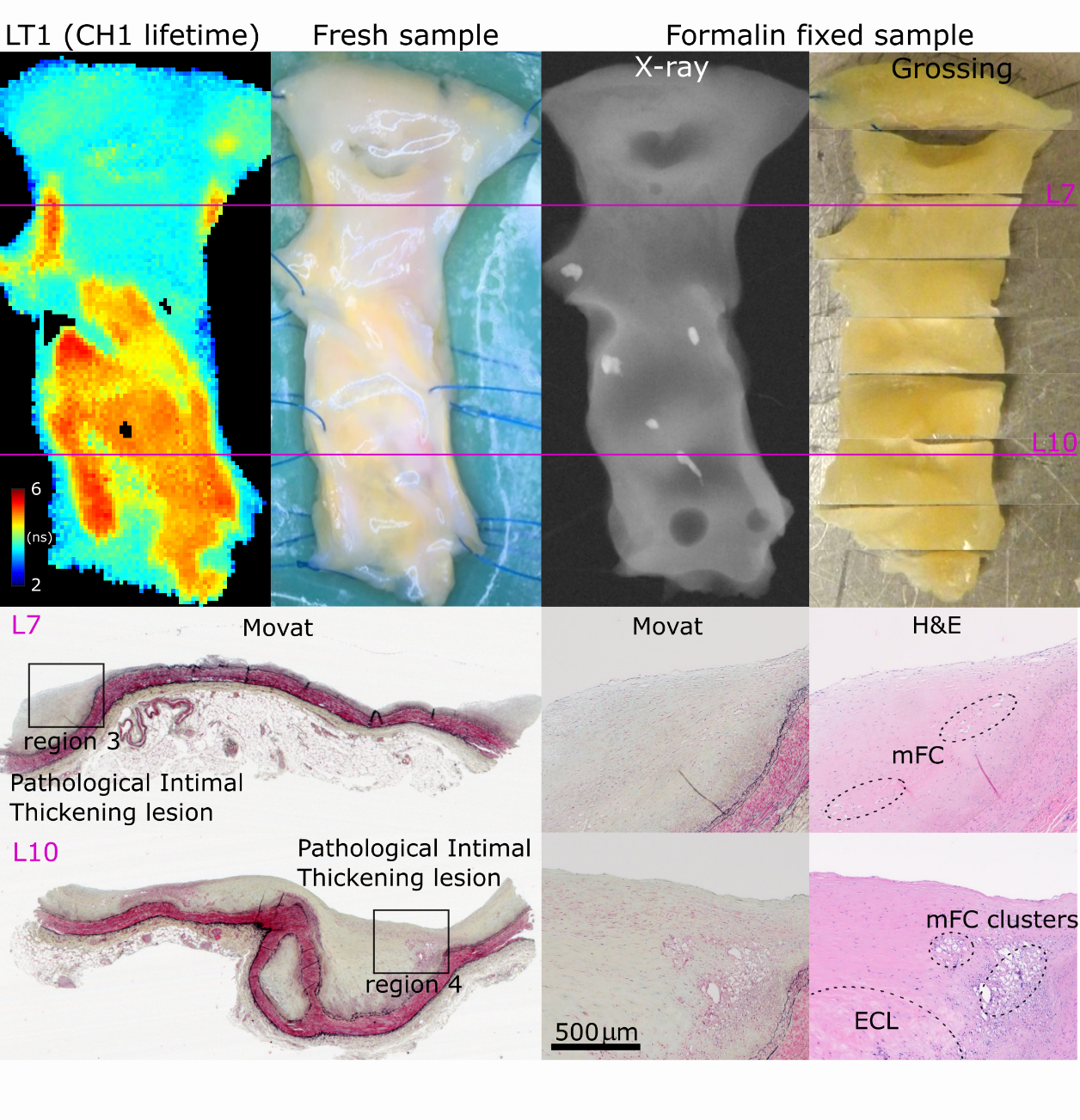
**

**Supplemental Figure 2: Overview of the imaging and histology processing. Detailed histology for sections L7 and L10 highlights healthy areas as well as atherosclerotic lesions characterized by the presence of macrophage foam cells (mFC) as well as calcifications (region 3: pathological intimal thickening), and ECL as well as mFC (region 4: PIT).**
